# Solid variant of aneurysmal bone cyst of the thoracic spine: a case report

**DOI:** 10.1186/1752-1947-5-261

**Published:** 2011-06-30

**Authors:** George Al-Shamy, Katherine Relyea, Adekunle Adesina, William E Whitehead, Daniel J Curry, Thomas G Luerssen, Andrew Jea

**Affiliations:** 1 Neuro-Spine Program, Division of Pediatric Neurosurgery, Texas Children's Hospital, Department of Neurosurgery, Baylor College of Medicine, Houston, TX, USA; 2 Department of Pathology, Texas Children's Hospital, Baylor College of Medicine, Houston, TX, USA

## Abstract

**Introduction:**

The solid variant of aneurysmal bone cyst is rare, and only 13 cases involving the spine have been reported to date, including seven in the thoracic vertebrae. The diagnosis is difficult to secure radiographically before biopsy or surgery.

**Case report:**

An 18-year-old Hispanic man presented to our facility with a one-year history of left chest pain without any significant neurological deficits. An MRI scan demonstrated a 6 cm diameter enhancing multi-cystic mass centered at the T6 vertebral body with involvement of the left proximal sixth rib and extension into the pleural cavity; the spinal cord was severely compressed with evidence of abnormal T2 signal changes. Our patient was taken to the operating room for a total spondylectomy of T6 with resection of the left sixth rib from a single-stage posterior-only approach. The vertebral column was reconstructed in a 360° manner with an expandable titanium cage and pedicle screw fixation. Histologically, the resected specimen showed predominant solid fibroblastic proliferation, with minor foci of reactive osteoid formation, an area of osteoclastic-like giant cells, and cyst-like areas filled with erythrocytes and focal hemorrhage, consistent with a predominantly solid variant of aneurysmal bone cyst. At 16 months after surgery, our patient remains neurologically intact with resolution of his chest and back pain.

**Conclusions:**

Because of its rarity, location, and radical treatment approach, we considered this case worthy of reporting. The solid variant of aneurysmal bone cyst is difficult to diagnose radiologically before biopsy or surgery, and we hope to remind other physicians that it should be included in the differential diagnosis of any lytic expansile destructive lesion of the spine.

## Introduction

Aneurysmal bone cyst is an expansile, non-neoplastic tumor-like lesion, commonly occurring around the knee and, rarely, in the vertebral column. Histologically, aneurysmal bone cyst is typically characterized by cavernous channels surrounded by a spindle cell stroma with osteoclast-like giant cells and osteoid production [1]. There is a distinct solid variant of aneurysmal bone cyst, first described by Sanerkin *et al*. [2] in 1983; the authors described four cases of an unusual intra-osseous fibroblastic lesion with scattered osteoclastic, osteoblastic, fibromyxoid elements, without a predominant component of cavernous channels. This solid variant may be easily misdiagnosed as a spindle cell tumor, especially osteosarcoma [3]. It is a rare lesion, accounting for 3.4% to 7.5% of all aneurysmal bone cysts [3], and only 13 cases [3,4] occurring in the spine have been previously reported. These cases have almost exclusively involved the pediatric age group, ranging in age from six to 17 years. Although the solid variant of aneurysmal bone cyst has the same biological nature as conventional aneurysmal bone cyst, the two forms differ in MRI scan findings.

We report a case of the solid variant of aneurysmal bone cyst occurring in the T6 vertebra with extensive involvement of the left sixth rib and pleural cavity in an 18-year-old Hispanic man. We review the 13 prior cases that have been reported in the literature and discuss the unique features of these unusual tumor-like lesions of the vertebral column.

## Case presentation

An 18-year-old, previously healthy Hispanic man presented to our institution with a one-year history of left paraspinal tenderness and radiation into the left chest. Our patient denied weakness or numbness of the legs and bowel or bladder incontinence. He had no difficulties with ambulation or balance.

On physical examination, tenderness could be elicited on palpation of the spinous processes of the mid-thoracic spine. No motor or sensory deficits were observed. There were no signs of myelopathy. A rectal examination showed good volitional rectal tone and no perineal anesthesia. The post-void residual volume of urine was negligible.

**Figure 1:**
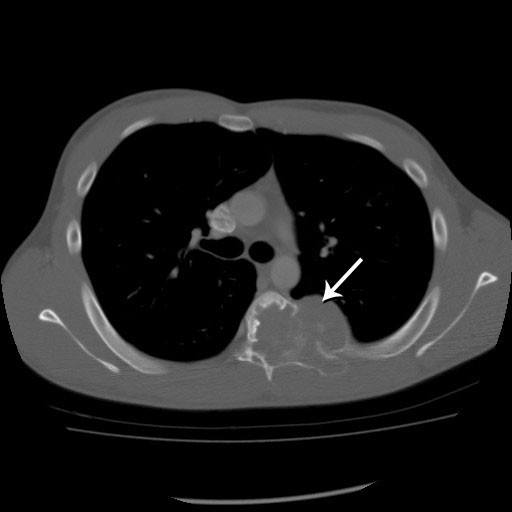
**Pre-operative axial computed tomography (CT) windowed for bone at the level of T6 shows an osteolytic and expansile lesion predominantly involving the left vertebral body, posterior elements, and proximal rib with a large intra-thoracic soft tissue component**.

**Figure 2:**
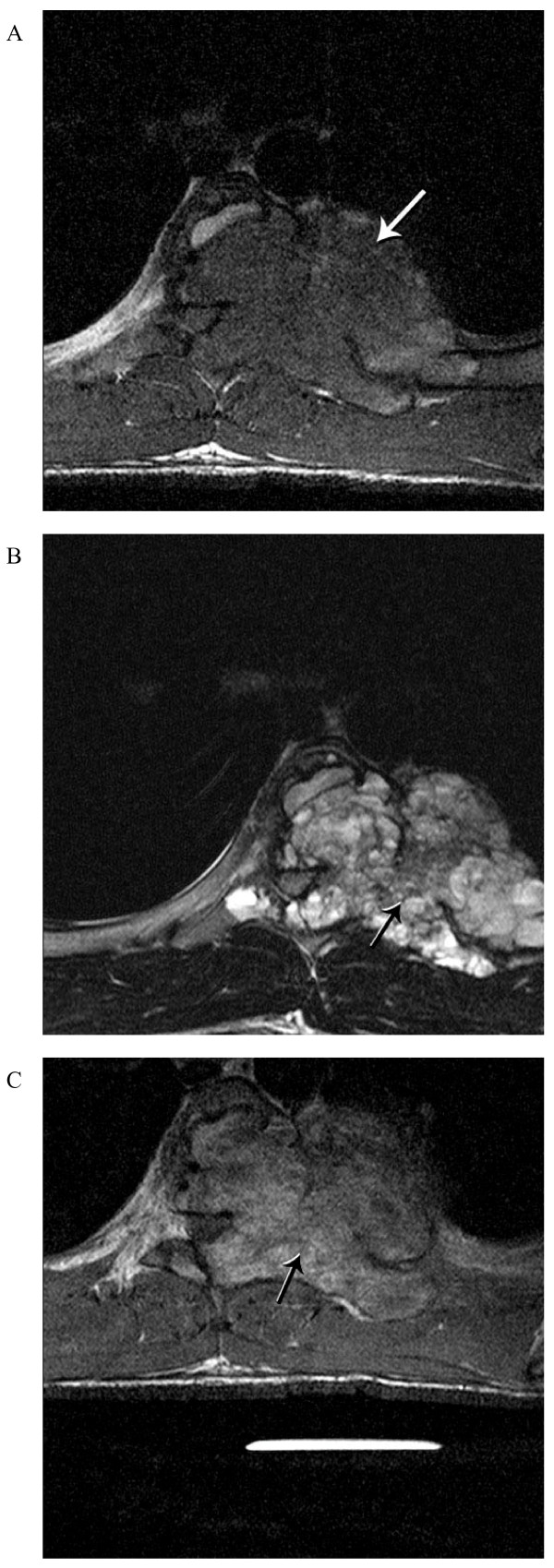
**Pre-operative axial (A) T1-weighted and (B) T2-weighted MRI demonstrate a large heterogeneous low and high signal intensity mass lesion involving T6**. (C) Enhanced T1-weighted MRI shows a more homogenous high signal T6 mass.

A computed tomography (CT) scan of the thoracic spine (Figure 1) demonstrated an expansile osteolytic lesion occupying the left part of the vertebral body of T6 destroying the lamina and pedicle as well as the associated rib at that level. MRI of the thoracic spine (Figure 2) revealed a large hypointense lesion on T1-weighted images with homogenous enhancement. The lesion showed mixed low-signal intensity with scattered high-signal intensity areas on T2-weighted MRI, suggesting microcysts.

Consideration was given to pre-operative spinal angiography and possible embolization of large arterial feeders to the mass. However, the risk of spinal cord infarction with embolization was deemed to be too high by the experienced interventional radiologists at our institution, and subsequently, this was not performed.

**Figure 3:**
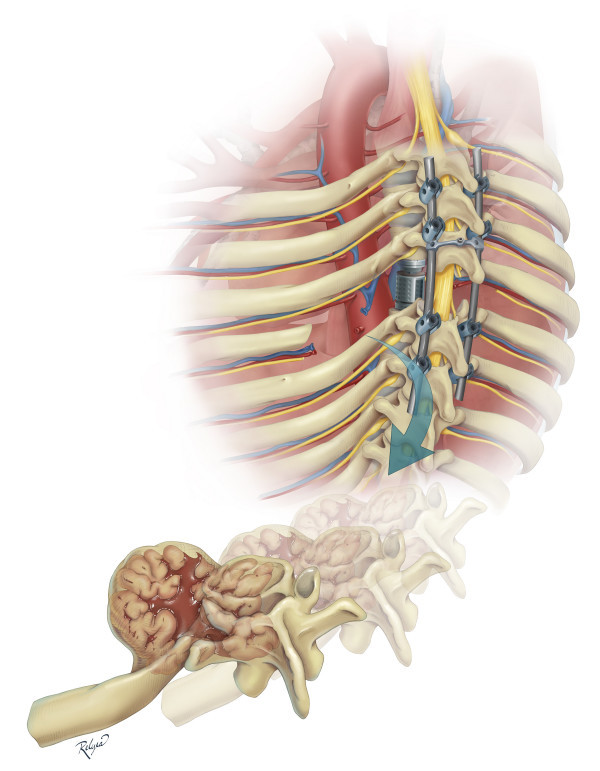
**Artist's illustration of the single stage posterior-only approach for resection of the tumor, left sixth rib, and T6 vertebral body with circumferential reconstruction of the spinal column**.

Our patient was taken to the operating room where a single stage posterior-only approach for total T6 spondylectomy with left sixth rib removal and circumferential reconstruction of vertebral column was planned (Figure 3). Spinal cord monitoring was performed with motor evoked potentials and somatosensory evoked potentials. A midline incision was made and a limb of the incision was extended toward the left, centered over T6 to provide a lateral extracavitary exposure. A T4 to T8 laminectomy was performed. Pedicle screws were placed at T4, T5, T7, and T8. After placing a temporary rod on the right side, the resection of the left sixth rib, mass lesion, and vertebral body of T6 proceeded in a piecemeal fashion. A section of parietal pleura was resected along with the tumor; there was no plane of separation between tumor capsule and pleura. A 13 mm diameter, 4° angle titanium expandable inter-body spacer spanned the T6 defect. An attempt was made to reduce the pre-operative kyphosis of 24° by compression between the pedicle screws at T5 and T7; however, there was a transient loss of motor evoked responses when this was performed. Therefore, the spine was fused *in situ *with no further attempts at correction of the kyphosis. Morselized bone graft from the osteotomized laminae and cancellous morselized allograft were used as graft material.

**Figure 4:**
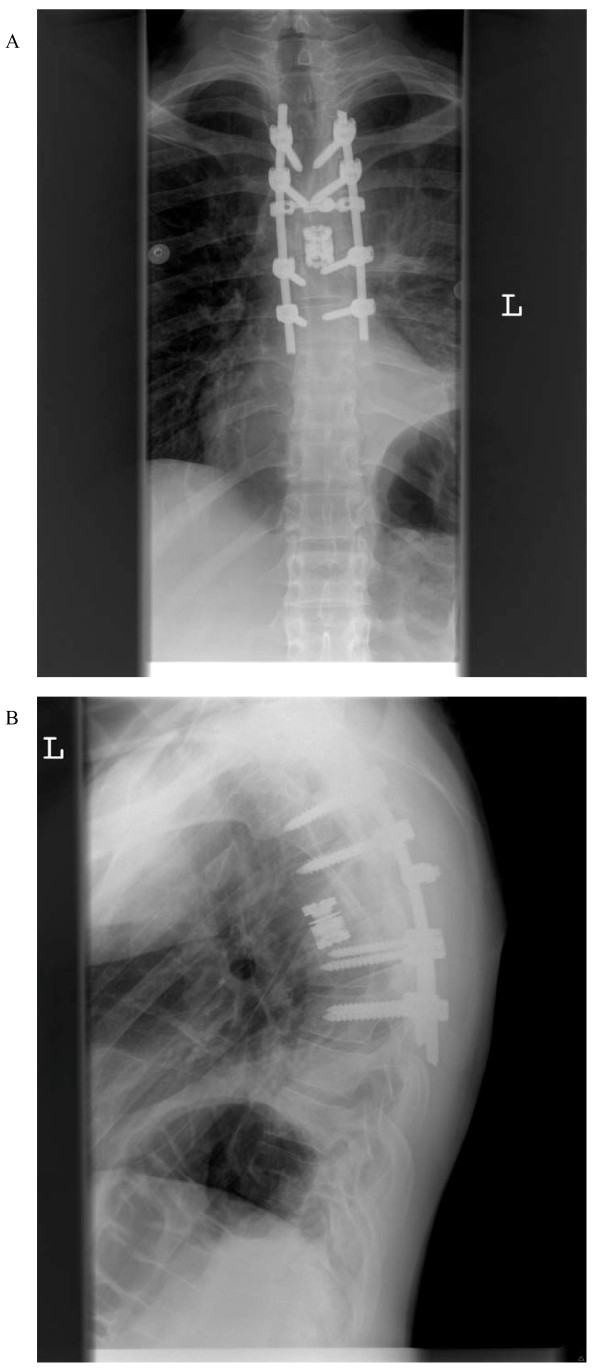
**Post-operative standing thoracic spine X-rays (A) AP and (B) lateral shows an expandable titanium cage filling T6 spondylectomy defect and posterior pedicle screw fixation**.

Post-operatively, our patient was neurologically intact. However, he did develop a pleural effusion on the left side on the first post-operative day that necessitated chest tube placement. The effusion was likely from irritation of the pleura and post-operative oozing of fluid/blood from the operative bed directly into the pleural cavity. There was no hematoma, and there were no signs of infection. After the chest tube was removed two days later, our patient progressed quickly in his recovery from surgery and was subsequently discharged home. Imaging revealed gross total resection of the mass lesion, adequate screw placement and moderate kyphosis of 34° (Figure 4). At 16 months after surgery our patient continues to do well and to be satisfied with the surgery, remaining pain free and neurologically intact and attaining radiological bony fusion without evidence of tumor recurrence.

**Figure 5:**
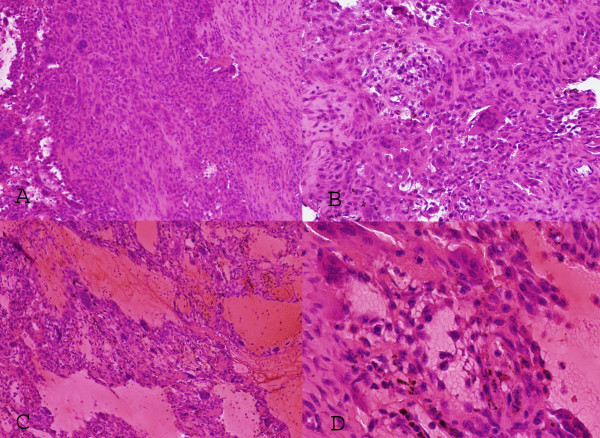
**Photomicrographs (A) (×100) and (B) (×200) illustrate the proliferating round to oval cells mixed with randomly distributed multi-nucleated giant cells**. Regions of reactive fibroblastic proliferation are present. Panels (C) (×200) and (D) (×400) show an example of region of tumor with the blood filled microcystic component.

Frozen and permanent sections (Figure 5) showed a predominantly solid lesion with frequent giant cells. The surrounding tissues included skeletal muscle, adipose tissue, and nerve bundles. The lesion consisted of oval to slightly spindled stromal cells interspersed with multi-nucleated osteoclast-like giant cells. There were areas of hemosiderin deposition, calcification, and reactive bone formation within the mass. Cyst-like areas filled with erythrocytes and areas of hemorrhage were also noted focally. No cytological atypia or brisk mitotic activity was appreciated. There was no evidence of malignancy. The histopathological features were consistent with those of a predominantly solid variant of aneurysmal bone cyst.

## Discussion

Aneurysmal bone cysts predominantly afflict children, with 60% of patients being younger than 20 years old; the peak incidence is during the second decade of life, and there is a slight preponderance for women over men [5,6]. In the same review of 94 cases by Hay *et al*. [6], the cervical spine was involved in 22% of cases, the thoracic spine in 34%, the lumbar spine in 31%, and the sacrum in 13%.

Bertoni *et al*. [3] reviewed 15 cases of the solid variant of aneurysmal bone cyst. The authors reported that the patient age distribution was two to 49 years (mean 23 years) and the male:female ratio was 1:1.5. The femur and tibia were the most commonly affected sites, and the spine was rarely affected.

Our review of 14 cases, including our patient, of spinal involvement of the solid variant of aneurysmal bone cyst is summarized in Table 1. The age of patients ranged from six to 18 years (mean, 11.4 years), and the male:female ratio was 1:1.8. More than half of the cases occurred in the thoracic spine. The cervical and lumbar vertebrae were involved in three cases each. Neck, back, or chest pain was the most common complaint on presentation. On average, symptoms persist for 12 months before definitive diagnosis. Conventional aneurysmal bone cyst of the vertebral column typically originates in the posterior neural arch and expands unilaterally to produce an eccentric paravertebral lesion [6]. In some cases of conventional aneurysmal bone cyst, destruction of the vertebral bodies with partial or complete collapse occurs.

**Table 1:** Previous reports of solid variant of aneurysmal bone cyst of the spine (modified from Suzuki *et al*. [10])

**Ref**.	Age	Sex	Site of lesion	Presenting signs and symptoms	Radiological findings	Treatment	Follow-up	Outcome
[2]	7	M	L4	Back pain, swelling, and abnormal gait	Expansile cystic lesion in L4 lamina	Tumor shelled out, laminectomy	six years	No recurrence

[2]	6	F	T2	Back pain and palpable tender mass	Destruction of lamina of T2	Partial piecemeal removal, laminectomy followed by irradiation (1.5 Gy)	one year	Residual mass

[2]	13	M	T7	Back pain, scoliosis, and myelopathy	Destruction of lamina of T7 with paravertebral mass	Subtotal excision, laminectomy, followed by irradiation (1.5 Gy)	three years	Recurrence at 6 months treated by curettage and bone graft with no recurrence for 3 years

[4]	10	F	C1	Pain and swelling	-	-	-	-

[7]	17	F	T1	Radiculopathy	Expansile lytic lesion in T1 lamina and spinous process	Subtotal excision, laminectomy	one year	No recurrence

[7]	16	F	T7	Back pain	Lytic lesion in T7 lamina and transverse process	Curettage and bone graft, irradiation (5 Gy)	eight years	No recurrence

[8]	9	F	L3	Back pain	Expansile osteolytic lesion in vertebral body, pedicle, transverse process, and lamina	Irradiation (20 Gy)	six years	No recurrence

[3]	14	F	C7	Neck pain	Expansile lytic lesion in spinous process of C7 and kyphotic deformity	N/A	N/A	N/A

[3]	8	M	L5	Radiculopathy	Expansile cystic lesion in L5 lamina and soft tissue mass causing L5 root compression	N/A	N/A	N/A

[3]	6	F	T2	Back pain	Destructive lytic lesion in T2 lamina and small rim of cortex in left paravertebral area	N/A	N/A	N/A

[3]	14	M	T7	Back pain	Destructive lytic lesion in T7 pedicle	N/A	N/A	N/A

[6]	12	F	T3-4	Back pain	Lytic lesion with destruction of neural arch	Excision and complete curettage	three years	No recurrence

[5]	9	F	C4	Neck pain	Expansile lytic lesion in C4 lamina and kyphotic deformity	Laminectomy, curettage followed by C2-5 fusion	one year	No recurrence

Our patient	18	M	T6	Chest pain	Expansile lytic lesion of T6 vertebral body, left pedicle, and lamina, and left sixth rib with soft tissue mass in left pleural cavity	Total spondylectomy T6 with left sixth rib resection and resection of intra-pleural soft tissue mass; circumferential reconstruction of vertebral column	16 months	No recurrence

The routine radiographic features on plain radiographs and CT of the solid variant of aneurysmal bone cyst include an osteolytic and expansile lesion that is indistinguishable from conventional aneurysmal bone cyst. Like conventional aneurysmal bone cysts, almost all cases reviewed of the solid variant aneurysmal bone cyst originated from the posterior elements of the vertebra. Involvement of the vertebral body, as in our patient, was rare and was reported in only two prior cases.

Similar to conventional aneurysmal bone cysts, MRI of the solid variant of aneurysmal bone cyst reveals homogeneous low-signal intensity on T1-weighted images and heterogeneous low-signal intensity with scattered high-signal intensity areas on T2-weighted images with possible fluid-fluid levels. This feature is very characteristic and highly suggestive of the diagnosis of aneurysmal bone cyst. In conventional aneurysmal bone cyst, thin, smooth septations of the lesion are seen in T1-weighted or T2-weighted images with contrast whereas enhanced MRI scans of the solid variant show more homogenous high signal intensity throughout the lesion. This is perhaps a distinguishing characteristic of solid aneurysmal bone cyst from conventional aneurysmal bone cyst.

Although these tumors are benign and spontaneous regression has been rarely described, prompt surgery appears to be the mainstay of treatment especially in cases of neurological compromise from nerve root or spinal cord compression, despite the lack of clear treatment guidelines. Most patients in our review were treated by a conservative attempt at curettage because of the benign character of these spinal lesions, although a higher rate of recurrence of up to 30% may develop after curettage [6]; therefore, the surgical goal should be a complete marginal excision. Radiation therapy was undertaken in two cases; reports of late post-irradiation sarcomas and post-irradiation myelopathy in patients with conventional aneurysmal bone cyst have made other authors more cautious about its use, and adjuvant radiation therapy should be reserved for patients with inoperable lesions because of location or associated medical conditions, or aggressive recurrent disease. Intra-cystic sclerosant injections, while favored in other locations, have resulted in mortality and major morbidities when used in the spine [7]. Embolization of feeding segmental arteries has been proposed as a pre-operative adjunct or sole treatment for aneurysmal bone cysts [8,9]; however, embolization as the sole mode of therapy has very limited applications in the spine, especially in the setting of pathological fracture and neurological compromise. In addition, embolization of multiple small feeding vessels is technically difficult, and inadvertent embolization of segmental arteries to the spinal cord may result in spinal cord infarction. Despite these concerns, the literature [10] suggests angiography and embolization can be performed without a significant risk of permanent neurological deficit, skin, or muscle necrosis. However, in our case, the experienced interventional neuroradiologists at our institution deemed the risk higher than usual given the proximity of the feeding artery to the tumor and the anterior spinal artery, combined with the watershed location at T6.

Depending on the proliferative component, the solid variant of aneurysmal bone cyst may be histologically misdiagnosed for other benign and malignant and tumor-like lesions of the bone. The pathological differential diagnosis includes solitary bone cyst, hemangioma, osteosarcoma, giant cell tumor, and chondroblastoma.

## Conclusions

Our patient was treated with an aggressive posterior-only surgical approach for complete resection of the aneurysmal bone cyst and circumferential reconstruction of the vertebral column with preservation of neurological function. Whether an aggressive surgical approach results in a better outcome and recurrence rate than a more conservative one (for example, curettage alone) remains to be seen in longer-term follow-up, and is the subject of future studies.

## Consent

Written informed consent was obtained from the patient for publication of this case report and any accompanying images. A copy of the written consent is available for review by the Editor-in-Chief of this journal.

## Competing interests

The authors declare that they have no competing interests.

## Authors' contributions

GA was responsible for the concept and design of the manuscript and for writing and editing of the manuscript. KR aided in the illustration of the manuscript. AA analyzed and interpreted the pathological data for our patient. WEW aided in the editing of the manuscript. DJC aided in the editing of the manuscript. TGL aided in the editing of the manuscript. AJ was responsible for the concept and design of the manuscript and for writing and/or editing the manuscript. All authors read and approved the final manuscript.
